# Sex‐specific benefits of energy supplementation in a rat model of Alzheimer's disease

**DOI:** 10.1002/alz70855_107444

**Published:** 2025-12-24

**Authors:** Mary Hill, Aaron Y Lai, Dustin Loren V Almanza, Jessica Ribeiro, Matthew Mandrozos, Bojana Stefanovic, JoAnne McLaurin

**Affiliations:** ^1^ Sunnybrook Research Institute, Toronto, ON, Canada; ^2^ Biological Sciences, Sunnybrook Research Institute, Toronto, ON, Canada

## Abstract

**Background:**

Consumption of high‐carbohydrate‐high‐fat diet leading to high body mass index either increases or decreases Alzheimer's disease (AD) risk with no clear consensus. We aim to differentiate the relationship between high energy diet and AD in a controlled rat model of AD during early symptomatic phase of the disease when the brain requires increased energy source as it attempts to compensate for neuronal loss.

**Method:**

We fed 9‐month‐old TgF344‐AD rats resembling early symptomatic phase of human AD a varied high‐carbohydrate‐high‐fat diet for 3‐ and 6‐months. We examined cognitive function using the Barnes Maze. Histological examination of neuronal density, amyloid, hyperphosphorylated tau, myelin and glia were examined.

**Result:**

Three‐month‐diet increased brain glucose metabolism. slowed cognitive decline, increased myelin/oligodendrocyte density in TgF344‐AD rats without affecting non‐transgenic rats; however, it also decreased neuronal density, and promoted deposition of amyloid‐beta plaques and tau inclusions. After 6 months on the high‐carbohydrate‐high‐fat diet, detrimental effects on density of neurons, amyloid‐beta plaques, and tau inclusions persisted while the beneficial effects on myelin, microglia, and cognitive functions remained albeit with a lower effect size.

**Conclusion:**

By examining the effect of sex, we found that effects of obesity were stronger in female than in male TgF344‐AD rats indicating that consumption of high energy diet during early symptomatic phase of AD is protective in females.

